# P23 Benchmarking voriconazole therapeutic drug monitoring in adults and paediatrics

**DOI:** 10.1093/jacamr/dlad143.027

**Published:** 2024-01-03

**Authors:** Yostina Ishak, Robert Oakley, Ting Yau

**Affiliations:** St George’s University Hospitals NHS Foundation Trust, London, UK; St George’s, University of London, London, UK; St George’s University Hospitals NHS Foundation Trust, London, UK; St George’s, University of London, London, UK; St George’s University Hospitals NHS Foundation Trust, London, UK

## Abstract

**Background:**

Voriconazole is the first-line agent for invasive aspergillosis and microbiology approved fungal infections. Voriconazole acts as both a substrate/inhibitor of several hepatic cytochrome P450 enzymes and demonstrates non-linear kinetics due to metabolism saturation. Inter-individual variability of voriconazole pharmacokinetics is high.^1^ Therapeutic drug monitoring (TDM) of trough plasma levels can mitigate treatment failure/toxicity. A therapeutic index between 1 and 5.5 mg/L is aimed for.^1^ An online protocol directs IV/oral dosing in line with British National Formulary guidance. An audit was conducted to benchmark protocol compliance.

**Standards:**

Total compliance to the following standards was investigated for all voriconazole prescriptions: (i) suitable indication; (ii) documented stop or review date; (iii) protocol prescribed loading and maintenance dose; (iv) dose adjustment for non-therapeutic voriconazole trough level; and (v) trough sampling between 4 and 7 days since initiation.

**Methods:**

Retrospective voriconazole dispensing data (April 2020–April 2022) was used to identify treated adult and paediatric patients. Correlating electronic prescribing and medicines administration (ePMA) system data was examined in line with standards. Patients receiving voriconazole prophylaxis or under 7 days total treatment were excluded from data collection. Descriptive statistics were used to describe patient characteristics and compliance with standards.

**Results:**

In total, 47 patients (31 adults and 16 children) were identified. The IQR of age was 48 years. Total compliance was obtained to standard (ii) only. Overall, 94% of patients’ prescriptions possessed an appropriate indication and 6% were clinically appropriate with unclear documented indications. A total of 66% received protocol directed loading and maintenance doses, 6% were prescribed appropriate loading doses, without maintenance doses, and 28% received an inappropriate loading/maintenance dose. Within 4 to 7 days since treatment initiation, 55% had therapeutic trough levels; 27% had supratherapeutic levels, but only 33% of doses were reduced, whereas when subtherapeutic trough levels were encountered in 18% of cases, 75% of doses were increased. Overall, 47% had plasma trough levels taken within the recommended period; 30% did not have a trough level ordered, whilst the remaining 23% had sampling taken before 4 days or after 7 days.

**Conclusions:**

Person and system interventions are required to improve pre-/post-prescription standards. Prescriber education focusing on indication documentation and recent patient body weight for dosing is required. Whereas an ePMA prescribing package incorporating protocol guidance, consecutive loading and maintenance doses, plus trough sampling orders should be trialled. Protocol integrated instructions on dosing adjustment as done for other TDM drugs should be explored. Occasional nursing omissions of both 12-hourly loading doses on Day 1 of treatment should be addressed with practice educators. Reliance on an external laboratory and third party to communicate trough sample results was associated with delays. This should be investigated further as a cause of dose adjustment issues. ePMA availability of samples, direct laboratory communication with prescribers/pharmacists and investigations into cost-effectiveness of internal laboratory assays should be considered. Interventions should be agreed with stakeholders and reaudit occur. Earlier sampling and dosing practices better representing local the patient population could be investigated further as part of an antimicrobial stewardship improvement project.
